# Antineutrophil cytoplasmic antibody-associated vasculitis in presence of positive antiphospholipid antibody: a case report 

**DOI:** 10.1186/s13256-022-03256-3

**Published:** 2022-01-24

**Authors:** Maassoumeh Akhlaghi Kalahroodi, Maryam Loghman, Mahsa Ramezanpoor, Reza Shahriarirad, Ehsan Rahmanian

**Affiliations:** 1 Rheumatology Research Center, Tehran University of Medical Sciences, Tehran, Iran; 2 Fellowship of Rheumatology, Shariati Hospital, Tehran University of Medical Sciences, Tehran, Iran; 3 Obstetrician and Gynecologist, Shiraz University of Medical Science, Shiraz, Iran; 4 Thoracic and Vascular Surgery Research Center, Shiraz University of Medical Sciences, Shiraz, Iran

**Keywords:** Vasculitis, Antineutrophil cytoplasmic antibody, Antiphospholipid syndrome, Case report, Vasculopathy, Antiphospholipid antibodies

## Abstract

**Background:**

Antineutrophil cytoplasmic antibody-associated vasculitis is dominated by inflammatory occlusion of small vessels, causing tissue ischemia in various organs. This disorder has rarely been associated with vasculopathy, such as antiphospholipid syndrome.

**Case presentation:**

We report a case of a 48-year-old Persian male presenting with distal digital gangrene along with inflammatory arthralgia. High titers of anti-proteinase 3 and antiphospholipid antibodies (anticardiolipin antibody) were detected in laboratory evaluation. Therefore, a diagnosis of antineutrophil cytoplasmic antibody-associated vasculitis and antiphospholipid syndrome was made and treated with anticoagulant along with monthly pulses of cyclophosphamide and a daily dose of 1 mg/kg prednisolone.

**Conclusion:**

Our case, along with other reports, illustrates that these two entities can coexist. Therefore, monitoring antiphospholipid antibodies in patients with antineutrophil cytoplasmic antibody-associated vasculitis with or without clinical evidence of any thrombosis and ruling out thrombosis in cases that do not respond to proper treatment of vasculitis may be relevant to prevent irreversible or fatal organ damage.

## Introduction

Antineutrophil cytoplasmic antibody (ANCA)-associated vasculitis is mainly distinguished by inflammatory occlusion of small vessels, inducing tissue ischemia in several vital organs such as the kidney; the ear, nose, and throat region; airways; central nervous system; and, occasionally, the digestive tract [1]. Antiphospholipid syndrome (APS or Hughes’ syndrome) is an autoimmune systemic disorder characterized by arterial, venous, or small vessel thrombosis [2]. This entity has been reported in other vasculitides such as Wegener’s granulomatosis [WG, or granulomatosis with polyangiitis (GPA)] [3], giant cell arteritis [4], Churg–Strauss syndrome [CSS, or eosinophilic granulomatosis with polyarteritis (EGPA)] [5], and polyarteritis nodosa (PAN) [6]. We report a case of ANCA-associated vasculitis along with positive antiphospholipid antibodies (aPL).

## Case presentation

The patient is a 48-year-old Persian male with repeated episodes of redness of eyes in the last nine years, without pain or irritation, which was diagnosed as scleritis by an ophthalmologist, and was administered prednisolone and azathioprine. After 1.5 years, follow-up was discontinued due to resolution of symptoms.

The patient had a 1-year history of pain in small joints of hands and foot, accompanied by morning stiffness (15 min). Joint pain was more prevalent in the wrists, finger interphalangeal (IP), shoulder, and elbow. He also reported positive history of malaise, with no history of photosensitivity, oral aphthae, ulcer, intestinal diseases, hair loss, Raynaud phenomenon, dryness of eyes or mouth, and skin lesions, upper limb claudication, weight loss, smoking, or any addiction. He also did not have hypertension, diabetes, and any history of thrombosis.

A preliminary diagnosis of rheumatoid arthritis was assumed for the patient by the rheumatologist, and the patient was administered a regimen of prednisolone (7.5 mg/day), methotrexate (10 mg/week), and hydroxychloroquine (200 mg/day), which resulted in a relative improvement of arthralgia symptoms.

**Fig. 1:**
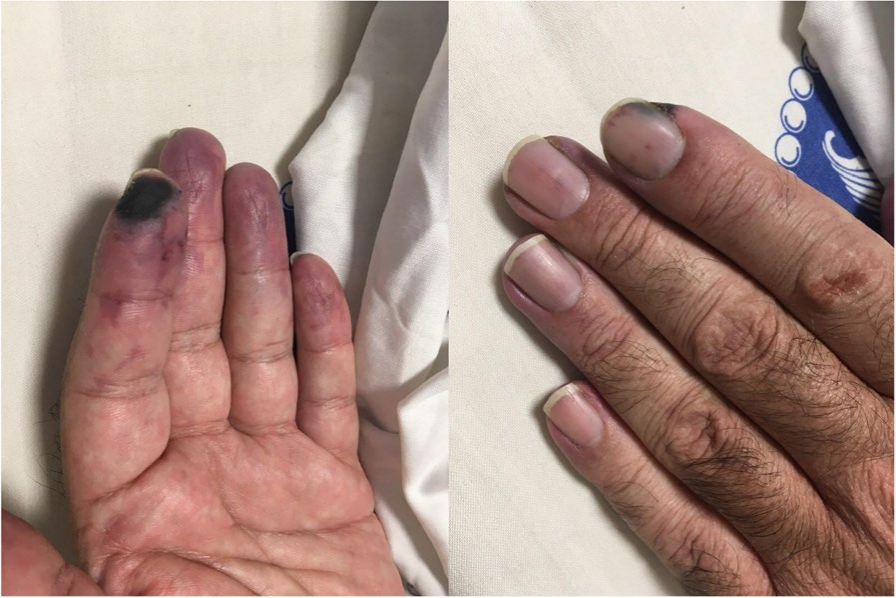
Gangrene of left index finger along with splinter hemorrhage under fingernails

After eight months of following the mentioned treatment regimen, the patient arbitrarily discontinued his drugs due to the COVID-19 pandemic. Consequently, he developed pain and paresthesia in his fingertips with cyanosis of the left index finger from 1 week before visiting us at the rheumatology clinic. On evaluation, there was evidence of cyanosis of the left index finder at its radial side, along with mottling signs and coldness at the distal part of the remaining fingers. Splinter hemorrhage was also observed under the fingernails (Fig. 1).

There was no evidence of arthritis, swelling, or tenderness in his wrist, interphalangeal, and metacarpophalangeal joints. Radial and ulnar pulses were present and symmetric in both hands. Heart and lung auscultation were normal with no signs of heart souffle. No evidence of bruit was detected in carotid, subclavian, abdominal, and renal artery examination. Abdominal and genitalia examinations were normal and without tenderness. Central and peripheral nervous system examinations were unremarkable.

Rheumatological laboratory evaluation demonstrated elevated rheumatoid factor levels with high titers, and cytoplasmic antineutrophil cytoplasmic antibodies (C-ANCA or anti-PR3) was ten times the normal value. Kidney and liver function tests were unremarkable, and the SARS-CoV-2 polymerase chain reaction (PCR) test was negative. Urine analysis was also unremarkable with no presence of cellular cast or protein (WBC: 2–3 and RBC: 0–3). Table 1 demonstrates the laboratory data of our patient.

**Table 1.** Laboratory data of 48-year-old patient with index finger cyanosis

Test	Reference value	Result	Interpretation
White blood cell count (× 10^9^/L)	3.5–9.5	9	Normal
Hemoglobin (g/L)	12–17.5	14.8	Normal
Mean corpuscular volume (fL)	82–92	87	Normal
Platelet count (mL)	150–450	329	Normal
Blood urea nitrogen (mg/dL)	7–20	10	Normal
Creatinine (mg/dL)	0.6–1.2	0.9	Normal
Aspartate aminotransferase (U/L)	15–40	18	Normal
Alanine aminotransferase (U/L)	9–50	19	Normal
24-h urine protein (mg/day)	< 80	126	Normal
Erythrocyte sedimentation rate (mm/h)	< 22	47	Elevated
C-reactive protein (mg/L)	0–8	67 (3+)	Elevated
Rheumatoid factor (IU/mL)	< 20	175 (3+)	Elevated
Anti-citrullinated protein antibody (IU/mL)	< 20	3.5	Negative
Antinuclear antibodies		1:100	Negative
Double-stranded DNA antibody (IU/mL)		–	Negative
Complement component 3 (mg/dL)	90–150	127	Normal
Complement component 4 (mg/dL)	10–40	17	Normal
Cytoplasmic antineutrophil cytoplasmic antibodies (AU/mL)	<15	113	Elevated
Perinuclear antineutrophil cytoplasmic antibodies (AU/mL)	< 15	< 3	Normal
Lupus anticoagulant		–	Negative
Anticardiolipin antibody IgG	< 30	65.2	Elevated
Anticardiolipin antibody IgM	< 30	22.6	Negative
Beta-2-glycoprotein I IgG (U/m)	< 40	4.8	Negative
Beta-2-glycoprotein I IgM (U/m)	< 40	< 3	Negative
Hepatitis B surface antigen (mIU)		–	Negative
Hepatitis C virus antibody (mIU)		–	Negative
Human immunodeficiency virus antibody (mIU)		–	Negative
SARS-CoV-2 PCR		–	Negative

**Fig. 2:**
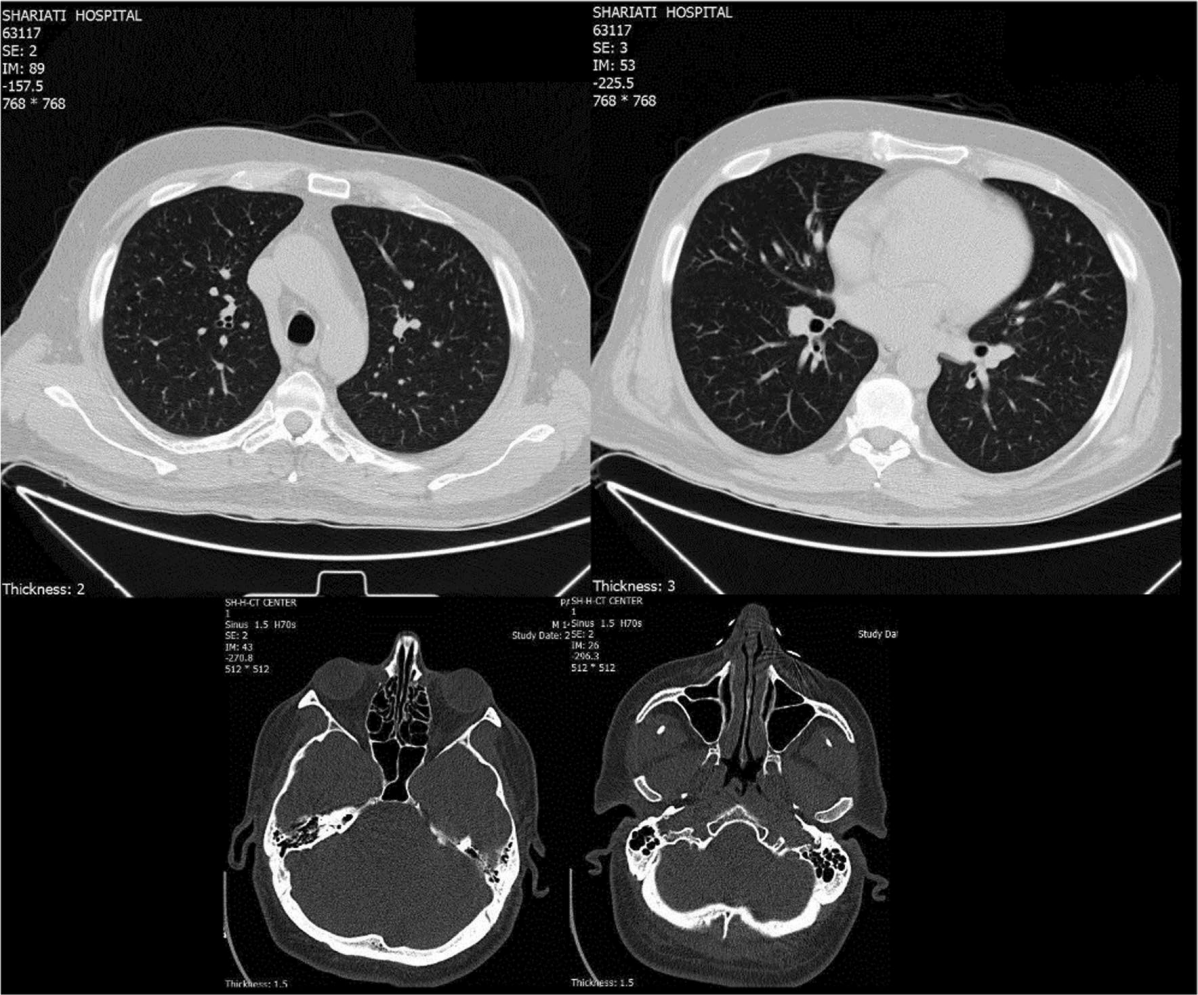
Normal computed tomography (CT) scan of lung and paranasal sinuses of 48-year-old male suspicious of vasculitis, with no evidence of alveolar hemorrhage, nodules, or cavitation

Electrocardiogram showed normal sinus rhythm. With suspicion of vascular obstruction, color Doppler sonography of the left upper limb artery and vein was performed, being normal with no signs of arterial stenosis or deep venous thrombosis. Cardiac echography was performed and revealed an ejection fraction of 55% and pulmonary artery pressure of 22, with no evidence of vegetation, mass, or thrombosis. Computed tomography (CT) scan of the lung and paranasal sinuses was normal with no evidence of alveolar hemorrhage, nodules, or cavitation (Fig. 2). Peripheral neurological evaluation through electromyography and nerve conduction velocity (EMG-NCV) test was unremarkable. The patient was also visited by an ophthalmologist, with no signs of uveitis and vasculitis detected, and sclera normal.

Based on a diagnosis of ANCA-associated vasculitis and obstruction of distal arteries of the small terminal artery of the extremities, infusion of methylprednisolone pulse (1 g/day) was started for 3 days, followed by venous pulses of cyclophosphamide (1 g/monthly) along with oral prednisolone (1 mg/kg/day).

During the course of treatment, he was still in pain, and the gangrene process continued. A positive value for aPL was detected, including a two-fold elevation of anticardiolipin (aCL) IgG levels. Therefore, anticoagulants were started for the patient through venous infusion of heparin (5000 unit/stat then 1000 U/h) along with oral administration of warfarin (5 mg/day). Furthermore, ASA (80 mg/day), diltiazem (30 mg, thrice a day), and atorvastatin (40 mg/day) were administered to the patient. Heparin infusion was halted after 3 days, while anticoagulant treatment was continued with warfarin (5 mg/day). The methylprednisolone 3 day course was followed with oral administration of 1 mg/kg prednisolone (30 mg, thrice a day).

**Fig. 3:**
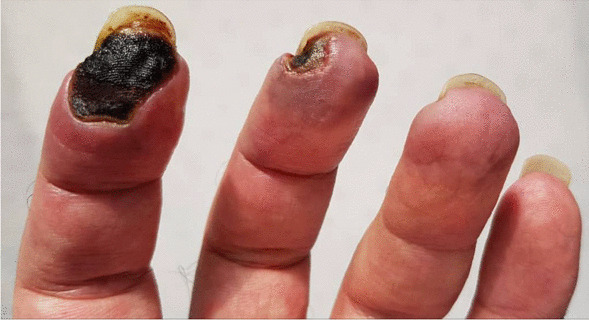
Limited gangrene of left index and middle finger in a 44-year-old patient with ANCA-associated vasculitis in presence of positive aPL, with blood flow around the mentioned areas

Laboratory data were repeated two weeks after finishing treatment. Anti-PR3 levels were still elevated (184 IU/mL). Erythrocyte sedimentation rate (2 mm/h) and C-reactive protein (2 mg/L) levels were within the normal range. Urine analysis demonstrated proteinuria, while 24-h urine showed 210 mg protein/day (creatinine 1170 mg, volume 3000 cc). Complete blood count and liver function test results were unremarkable. The patient was discharged 5 days of admission with improved symptoms during follow-up. We repeated the aCL tests during the patients third month of follow-up, which again demonstrated positive ACL antibodies (aCL Ab (IgG) = 51.5; NL < 30), confirming our previous diagnosis of APS. During follow-up and examination, gangrene stopped and limited to the area at early points of diagnosis. There was also sufficient blood flow around the mentioned area (Fig. 3).

## Discussion

We present herein a middle-aged male patient with ANCA-associated vasculitis in presence of positive aPL. He had previously received prednisolone and azathioprine due to scleritis, and also prednisolone, methotrexate, and hydroxychloroquine, with suspicion of rheumatic arthritis, which he arbitrarily discontinued. This was followed by index finger gangrene along with paresthesia and mottling sign of the other fingers. The first assumption was vasculitis, in regard to which pulses were present and normal with unremarkable color Doppler sonography, which raised the suspicion of medium and small vessel involvement.

Two primary diagnoses were considered for the patient:

1. Vasculitis: based on his history of scleritis, inflammatory arthritis, suspicious of rheumatologic diseases such as rheumatoid arthritis (RA), systemic lupus erythematosus (SLE), and even medium-sized artery vasculitis such as PAN, ANCA-associated vasculitis such as GPA, and also Buerger’s disease.
2. Vasculopathy: such as arterial thrombosis, cholesterol emboli, atrial myxoma embolism, and antiphospholipid syndrome (APS).

We treated the patient on the basis of a possible preliminary diagnosis of ANCA-associated vasculitis, although the criterion for a critique of vasculitis was not met since we did not have a tissue biopsy as all of our organ evaluations were normal. Since some clinical settings, such as digital ischemia or scleritis, do not require biopsy if the anti-pR3 test is positive [7], we started a 3-day course of corticosteroid pulse with suspicion of vasculitis, followed by induction of a monthly venous pulse of cyclophosphamide. Based on the positive high levels of anti-PR3 in the patient, other vasculitides were assessed. Color Doppler sonography was performed to rule out large vessel involvements, being unremarkable. Vasculitides involving paranasal sinuses and the lung, such as GPA, were ruled out through CT evaluation. EMG-NCV was performed for neurological assessment of peripheral nerves based on vasculitis such as polyarthritis nodosa, also being normal. Cardiac assessment was performed to rule out bacterial endocarditis, atrial myxoma, and thrombosis.

However, while the patient was treated as a case of vasculitis, there was progression in the gangrene, which also started in his middle finger. On further evaluation, aPL was also positive (aCL IgG antibody), with two times the normal value, while SLE tests were negative. With positive levels of aPL, and continuing gangrene process, there was a possibility of thrombosis in the finger arteries. We could not differentiate between vasculitis and vasculopathy, so anticoagulants were also administered based on positive aPL levels and presence of tissue gangrene. Therefore, APS was considered for the patient alongside ANCA-associated vasculitis. Accordingly, the treatment regimen consisted of anticoagulant (warfarin 5 mg/daily) and monthly pulses of cyclophosphamide and a daily dose of 1mg/kg prednisolone. The matter of debate is whether aPL tests can be simultaneously positive with vasculitis, especially ANCA-associated vasculitis. This phenomenon has rarely been reported.

APS in presence of vasculitis has been reported in GPA [3], giant cell arteritis [4], EGPA [5], and PAN [6]. The current case, along with other reports, illustrates that these two entities can coexist. Therefore, in evaluating the need for prophylactic anticoagulation for thrombosis prevention, monitoring antiphospholipid antibodies in patients with vasculitis with clinical evidence of internal organ thrombosis may be relevant.

In a study by Sebastian* et al*., 21 of 176 (12%) patients with GPA had aCL, including 3 of 29 (10%) patients with thrombosis [8]. Rees* et al*. also reported a prevalence of 17% aPL in primary systemic vasculitis [9]. Weider *et al*. reported 13 patients who presented with venous thromboembolism (VTE) at time of diagnosis of active ANCA-associated vasculitis [1].

Ferenczi *et al*. [5] reported a case of EGPA in which severe digital gangrene developed in addition to cutaneous vasculitis. Although our patient did not exhibit the hallmarks of EGPA, a similar presentation was observed since, in both studies, the patient developed digital gangrene due to vaso-occlusion in presence of positive aPL. Microthrombosis and vascular occlusion are usually linked with lupus anticoagulant with or without aCL antibodies rather than genuine leukocytoclastic vasculitis. The simultaneous presence of vasculitis and aPL in the current patient may have contributed to development of distal digital gangrene.

In autoimmune disorders, aPL may be aimed at phospholipids or b2-GP-I-associated phospholipids [10]. Interestingly, b2-GP-I adheres to the surface of endothelial cells, and b2-GP-I reactive antibodies may identify the b2-GP-I endothelial cell-bound complex, resulting in different biological effects, such as upregulation of adhesion molecules and proinflammatory secretion [11]. Hence, although neither sensitive nor specific to systemic vasculitis, these antibodies may play a pathophysiological role in the disease. The antibodies can also lead to vasculitis pathology if present, by affecting the thrombosis mechanism on the compromised endothelium [12].

Also, the absence of involvement of other organs such as kidney or lung does not undermine the diagnosis of ANCA vasculitis since the patient may still develop these features in the future. Although our patient had no history of thrombosis, his first attack may present as digital gangrene. The patient has history of collagen and vascular disease (arthritis, scleritis), and now that he developed gangrene, a positive anti-PR3 test was detected. When the gangrene of the limb is positive with anti-PR3, we do not require a tissue biopsy and the diagnosis of ANCA-associated vasculitis is made. Therefore, we treated both vasculitis (cyclophosphamide) and antiphospholipid (anticoagulant) [7].

## Conclusion

aCL is present in many disorders included in the differential diagnosis of a patient suspected of having systemic vasculitis. We recommend that they be observed in vasculitis associated with ANCA because they can lead to vascular damage superimposed on life-threatening thrombotic events.

## Data Availability

All data regarding this case has been reported in the manuscript. Please contact the corresponding author if you are interested in any further information.

## Abbreviations

ANCA: Antineutrophil cytoplasmic antibody
aPL: Antiphospholipid antibodies
APS: Antiphospholipid syndrome
CT: Computed tomography
COVID-19: Coronavirus disease 2019
C-ANCA: Cytoplasmic antineutrophil cytoplasmic antibodies
EGPA: Eosinophilic granulomatosis with polyarteritis
EMG-NCV: Electromyography and nerve conduction velocity
GPA: Granulomatosis with polyangiitis
IP: Interphalangeal
PAN: Polyarteritis nodosa
RA: Rheumatoid arthritis
SLE: Systemic lupus erythematosus
WG: Wegener’s granulomatosis
